# NPM1 as a potential therapeutic target for atypical teratoid/rhabdoid tumors

**DOI:** 10.1186/s12885-019-6044-z

**Published:** 2019-08-28

**Authors:** Ji Hoon Phi, Choong-Hyun Sun, Se-Hoon Lee, Seungmook Lee, Inho Park, Seung Ah Choi, Sung-Hye Park, Ji Yeoun Lee, Kyu-Chang Wang, Seung-Ki Kim, Hongseok Yun, Chul-Kee Park

**Affiliations:** 1 Division of Pediatric Neurosurgery, Pediatric Clinical Neuroscience Center, Seoul National University Children’s Hospital, 101 Daehak-ro, Jongno-gu, Seoul, 110-744 Republic of Korea; 2 Department of Neurosurgery, Seoul National University College of Medicine, 101 Daehak-ro Jongno-gu, Seoul, 110-744 Republic of Korea; 3 Genome opinion Co., Ltd. 7, Yeonmujang 5ga-gil, Seongdong-gu, Seoul Republic of Korea; 4 Division of Hematology-Oncology, Department of Medicine, Samsung Medical Center, Sungkyunkwan University School of Medicine, Seoul, Republic of Korea; 5 Department of Health Sciences and Technology, SAIHST, Sungkyunkwan University School of Medicine, Seoul, Republic of Korea; 6 Graduated, Department of Statistics, Seoul National University, Seoul, Republic of Korea; 7 SD Genomics, Seoul, Republic of Korea; 8 Department of Pathology, Seoul National University College of Medicine, Seoul, Republic of Korea; 9 Department of Anatomy, Seoul National University College of Medicine, Seoul, Republic of Korea; 10 Center for Precision Medicine, Seoul National University Hospital, 101 Daehak-ro Jongno-gu, Seoul, 110-744 Republic of Korea

**Keywords:** Atypical teratoid/rhabdoid tumor, NPM1, Nucleophosmin

## Abstract

**Background:**

Atypical teratoid/rhabdoid tumors (AT/RTs) are highly malignant brain tumors with inactivation of the *SMARCB1* gene, which play a critical role in genomic transcriptional control. In this study, we analyzed the genomic and transcriptomic profiles of human AT/RTs to discover new druggable targets.

**Methods:**

Multiplanar sequencing analyses, including whole exome sequencing (WES), single nucleotide polymorphism (SNP) arrays, array comparative genomic hybridization (aCGH), and whole transcriptome sequencing (RNA-Seq), were performed on 4 AT/RT tissues. Validation of a druggable target was conducted using AT/RT cell lines.

**Results:**

WES revealed that the AT/RT genome is extremely stable except for the inactivation of *SMARCB1*. However, we identified 897 significantly upregulated genes and 523 significantly downregulated genes identified using RNA-Seq, indicating that the transcriptional profiles of the AT/RT tissues changed substantially. Gene set enrichment assays revealed genes related to the canonical pathways of cancers, and nucleophosmin (NPM1) was the most significantly upregulated gene in the AT/RT samples. An NPM1 inhibitor (NSC348884) effectively suppressed the viability of 7 AT/RT cell lines. Network analyses showed that genes associated with NPM1 are mainly involved in cell cycle regulation. Upon treatment with an NPM1 inhibitor, cell cycle arrest at G1 phase was observed in AT/RT cells.

**Conclusions:**

We propose that NPM1 is a novel therapeutic target for AT/RTs.

**Electronic supplementary material:**

The online version of this article (10.1186/s12885-019-6044-z) contains supplementary material, which is available to authorized users.

## Background

Malignant rhabdoid tumor is a highly aggressive neoplasm of early childhood that develops in the brain, kidney, and soft tissues. In particular, malignant rhabdoid tumors arising in the brain are called atypical teratoid/rhabdoid tumors (AT/RTs). The prognosis of an AT/RT is quite poor, as the 3-year overall survival rate is 22%, with an event-free survival rate of 13%. [1] Long-term survival is attained in less than 20% of patients [1, 2]. An improved 2-year overall survival rate of 60% or 70% has been reported; however, potentially toxic irradiation was applied to young infants in these studies [3, 4].

Malignant rhabdoid tumors, including AT/RTs, are characterized by genetic alterations affecting the *SMARCB1* (also known as *hSNF5* and *INI1*) locus in chromosome 22q11.2 or, rarely, the *SMARCA4* locus in chromosome 19p13.2 [5]. The SMARCB1 protein is a core subunit of the switch/sucrose non-fermentable chromatin remodeling (SWI/SNF) complex that regulates the expression of thousands of genes [6]. High-resolution genomic analysis and whole exome sequencing studies identified extremely low mutation rates for genes other than the biallelic inactivation of *SMARCB1* in rhabdoid tumors [5, 7]. A recent study in which whole genome sequencing was undertaken for many AT/RT samples also reported rare recurrent mutations aside from the mutations in *SMARCB1* [8]. Indeed, pediatric malignant rhabdoid tumors have the least number of somatic mutations among diverse human cancers [9]. These studies demonstrated that unlike other malignant cancers, the genome of the AT/RT is extremely stable, and alterations of SMARCB1 function must contribute to the numerous cancer hallmarks observed in the AT/RTs. Recent studies on the transcriptome and methylome of AT/RT have revealed that AT/RT can be divided into distinct molecular subgroups [10–12]. These subgroups are distinguished by epigenetic differences, reflecting the lack of driver oncogenes in AT/RT [10]. Distinct activation patterns of enhancers also characterize the molecular subgroups [8]. The pathogenesis of AT/RT with *SMARCB1* inactivation has not been fully elucidated. In a murine model, knockout of *SMARCB1* led to rhabdoid tumors in soft tissues but never in the brain [13]. Co-deletion of *SMARCB1* and *TP53* in mice caused rhabdoid tumor development in the brain reminiscent of AT/RTs [14]. However, concurrent *SMARCB1* and *TP53* deletions are rarely found in human AT/RTs. To date, no oncogenic driver mutation in a canonical pathway has been found in a human AT/RT [15]. The absence of an oncogenic driver mutation and a heterogeneous subclass of genomic characteristics make it difficult to find druggable targets for AT/RTs [8, 10, 11].

SMARCB1, along with other members in the SWI/SNF chromatin remodeling complex, regulates gene expression and DNA repair. The action of SMARCB1 is context-dependent and involves multiple cellular functions, such as the cell cycle, differentiation and cell survival [16]. At least one-third of genes in the genome are regulated by the SWI/SNF complex [17]. Transcriptional profiling showed that CCND1 and EZH2 overexpression may play an important role in rhabdoid tumors [18–20]. Inhibition of EZH2 impaired cell growth and abrogated tumorigenesis in mice [18, 21]. EZH2 is a component of the polycomb-repressive complex 2 (PRC2), and increased EZH2 expression is associated with the suppression of various genes through specific histone methylation. A recent study indicated that inactivation of *SMARCB1* leads to disruptions of specific nucleosome patterning and a loss of overall nucleosome occupancy at many promoters, generating altered genome-wide transcriptional levels [22]. Therefore, despite the lack of somatic mutations in AT/RTs other than *SMARCB1* inactivation, the extent and depth of transcriptional dysregulation are comparatively high. Among the many deregulated genes, identification of the most effective druggable target is still indispensable.

In this study, we sought to investigate the gene expression profiles of the AT/RT tissues that have biallelic inactivation of the *SMARCB1* gene. From the integrated genomic data from tumor samples of 4 AT/RT patients, we narrowed down the candidate therapeutic targets to identify nucleophosmin (NPM1), a multifunctional phosphoprotein involved in ARF/p53 pathway regulation, as a novel potential therapeutic target for AT/RTs. In vitro experiments using AT/RT cell lines confirmed the anticancer effect of NPM1 inhibition.

## Methods

### Patient samples

Pairs of snap-frozen tumor tissues and matched normal blood samples collected at the time of surgery from 4 patients who were histologically diagnosed with an AT/RT were used for DNA and RNA extraction. All patients had tumors in their cerebellum and were infants or of early childhood age (3–18 months). Clinical information is summarized in Additional file 1: Table S1. Genomic DNA was extracted using a QIAamp DNA mini kit (Qiagen, Cat. No. 51304), and total RNA was extracted using an RNeasy Plus Universal Mini Kit (Qiagen, Valencia, CA, USA, Cat. No. 73404) according to the manufacturer’s recommendations. DNA content was quantified using a Qubit DNA quantification kit (Invitrogen, Carlsbad, CA), and DNA integrity was assessed by gel electrophoresis. Samples with an RNA Integrity Number (RIN) > 5 were selected for the study. Whole exome sequencing (WES), single nucleotide polymorphism (SNP) arrays, array comparative genomic hybridization (aCGH), and whole transcriptome sequencing (RNA-Seq) were performed using these samples, except for the normal blood sample of one patient (P2) due to poor DNA quality. Two AT/RT tissues used for WES were available for confirmation in qPCR in which 8 additional AT/RT tissues were utilized. Medulloblastoma tissues (*N* = 5) were used for comparison. This study was approved by the Institutional Review Committee of the Seoul National University Hospital, and written informed consent was obtained from all patients for the usage of the samples. We excluded all identifying information of the participants from this article.

### Whole exome sequencing

We used an Agilent SureSelect 50-Mb exome capture kit (Agilent Technologies Inc., Santa Clara, CA) for exon target enrichment. Sequencing was performed using an Illumina HiSeq 2000 system with 100-bp paired-end reads. Using hg19 as a reference genome, mapping and paring were performed using the BWA algorithm. Local realignment was performed using GATK (http://www.broadinstitute.org/gatk/), and duplication removal was conducted using Picard. We obtained final BAM files with more than 150 times the depth of coverage on the target for all samples except one matched normal blood sample (P2) in which the DNA sample quality was too poor to be sequenced. The details of the calling process for single nucleotide variants (SNVs) and indels are described in the Additional files 1 and 2. Using SnpEff (ver3.6, http://snpeff.sourceforge.net), we selected variations that were non-synonymous, and rarity in the general population was defined as < 1% in the one-thousand genome project (http://www.1000genomes.org/) [23, 24].

To call SNVs and small indels from the WES data, we used the Unified Genotyper (GATK-Lite 2.3.9) on the BAM files that resulted from preprocessing of the lane-level BAM files with the protocol described in the “Best Practice Variant Detection with GATK v4”. Among the protocols described in that practice, we followed the “Best: per-sample realignment with known indels then recalibration” protocol for the tumor/normal contrastive calling projects. To produce lane-level BAM files from raw sequence reads, we mapped the raw sequence reads of each lane to hg19 using the BWA algorithm. For each variant that was called with the Unified Genotyper, we selected tumor-specific variants with the following somatic variant calling protocol, which consisted of 3 steps, as follows. 1) We separately counted the number of high-quality reads supporting the reference allele and the variant allele for each variant site. 2) We performed a permutation test that was designed to determine whether a variant allele fraction (VAF) in a tumor sample was higher than that of the normal sample. At this step, we calculated the VAF from the read count of the reference allele and that of the variant allele for each sample. 3) Finally, we selected somatic variants considering the *p*-value from the permutation test and VAF_frac_ (VAF_Tumor_/(VAF_Tumor_ + VAF_Control_)) together. We used VAF_frac_ to denote a ratio between a VAF of the tumor sample and the sum of VAFs of each sample. For this purpose, we used a volcano plot between VAF_frac_ and –log_10 (*p*-value). During somatic variant calling, we excluded low-quality variant sites that were determined based on the depth of high-quality reads and other properties of the variant site.

### Copy number alteration analysis

For copy number alteration (CNA) analysis of the whole exome capture sequencing, we used CONTRA (ver1.0, http://contra-cnv.sourceforge.net/) [25]. The Agilent SureSelect 50 M bed file was used as the target region definition file and was applied to the CNA analysis. The final results were summarized as the exon level log2-fold changes of read depth between the normal and tumor samples into the gene-level log2-fold changes using an in-house script. Loss of heterozygosity (LOH, heterozygous in the normal tissue, but homozygous in the tumor tissue) was defined using VAF values of the normal and tumor samples.

### Whole transcriptome sequencing

The 200~500 bp double-stranded cDNA fragments were purified by agarose gel electrophoresis and amplified using PCR to produce libraries. Raw sequencing reads were produced using Illumina HiSeq 2000 with 100-bp paired-end reads. After removing poor-quality raw reads containing the adaptor sequence, more than 10% of unknown bases or low quality bases, the remaining reads were aligned to the human reference genome (hg19). Expression profiles were analyzed using the reads per kilobase per million mapped reads (RPKM) values. Details of the expression quantification process are described in the Additional files 1 and 2.

### Differentially expressed gene analysis

To perform the differentially expressed gene (DEG) analysis, we used open source RNA-Seq data of normal brains (BrainSpan, http://www.brainspan.org) as a control. Five age-matched cerebellar cortex RNA-Seq datasets from the BrainSpan database were selected (Additional file 1: Table S2). The expression quantification process we employed was identical to that of BrainSpan. Alignment of the reads was performed using TopHat (version 1.3.1) [26]. For the human genome mapping, the GTF format annotation file, Gencode version 10 (GRCh37, Ensembl 65), was additionally provided to improve the mapping quality of exon-exon junction reads. After alignment, SAMtools (http://samtools.sourceforge.net/) and RSEQtools (https://github.com/gersteinlab/RSEQtools) software packages were used to perform RPKM-based quantification as described. RPKM values were computed using the “mrfQuantifier” program in RSEQtools. For the gene model, the gene composite model that is generated using the GTF format annotation file (Gencode version 10) was used.

Differentially expressed genes were analyzed with the DESeq package in R software (version 3.0.2; http://www.r-project.org/) using the negative binomial test [27]. Up- and downregulated genes were identified as those with RPKM values > 0.5 and with *p*-values < 0.05. With the use of publicly available data such as BrainSpan, batch effects between our data and the BrainSpan data are inevitable. However, RPKM quantification, which normalizes raw read counts to regions within a sample, may eliminate batch effects. Additionally, to select differentially expressed genes, we considered prior knowledge from two whole genome expression studies on a microarray platform after *SMARCB1* inactivation in mouse embryonic fibroblasts (MEFs) [22, 28]. These studies suggested that mammalian SMARCB1 (or SWI/SNF complex) may act to repress transcriptional activation rather than to activate transcriptional repression, and the ratio of up/downregulated genes was approximately 1.5~3. DEG analysis between the 4 AT/RT datasets and the 5 BrainSpan datasets was performed in R with the SAMSeq (Wilcoxon rank test) and DESeq packages (negative binomial test), respectively. The negative binomial test computed statistically significant p-values for each gene in Gencode version 10 (GRCh37, Ensembl 65).

### Single nucleotide polymorphism arrays

We applied a genome-wide SNP array (Illumina HumanOmni5-Quad BeadChip, Illumina) that covers 4,301,332 SNPs using genomic DNA samples. B allele frequency (BAF) values computed from the GenomeStudio software (Illumina) were exported, and the paired parent specific circular binary segmentation (PSCBS) method was used for LOH and CNA analysis. SNP array data were further analyzed using the ASCAT tool to estimate the tumor purity (Additional file 2: Figure S1) [3]. The estimated tumor purity and further results are summarized in Additional file 1: Table S3.

### Array comparative genomic hybridization

We used Agilent SurePrint G3 Human CGH Microarray 1 × 1 M arrays (Agilent Technologies) for aCGH analysis with tumor and matched normal genomic DNA samples. Raw data were acquired and normalized (LOWESS algorithm) using Feature Extraction software (v10.7, Agilent software). The significance test for each CNV region considered the Z-statistic using DNA Analytics (ver4.0.81, Agilent software), which sets the window size to 1 M and the Z-score threshold to 4.0.

### Gene set enrichment analysis

We performed a gene set enrichment analysis (GSEA) with the up/downregulated DEGs in the AT/RT samples [29]. DEGs were enriched into the predefined functional modules in GSEA (http://software.broadinstitute.org/gsea/msigdb/index.jsp) [30]. To select the cancer-associated genes in the gene sets that were significantly enriched, we employed the cancer class of the Genetic Association Database (GAD, https://geneticassociationdb.nih.gov) in DAVID (https://david.ncifcrf.gov). In addition, for the network analysis, we applied GeneMANIA software (version 3.1,2,8) [31].

### Cell lines and reagents

AT/RT cell lines (BT-12 and BT-16) were obtained from Dr. Peter Houghton (Nationwide Children’s Hospital). Primary cultured AT/RT cells were derived from 8 pediatric AT/RT patients (SNUH AT/RT 1~7, 10) as described previously [32]. The cells were maintained in DMEM (Gibco, Waltham, MA, USA) supplemented with 10% fetal bovine serum (FBS) (Gibco), penicillin (100 U/mL), and streptomycin (100 mg/mL) (Invitrogen, Waltham, MA, USA) in a humidified incubator at 37 °C and 5% CO_2_.

### Cell viability analysis

Cell viability was determined using the EZ-Cytox kit (iTSBiO, Seoul, Korea) according to the manufacturer’s protocol. The cells were seeded overnight at a density of 4000 cells per well in 96-well plates. Then, the cells were treated with an NPM1 inhibitor (NSC348884, Santa Cruz Biotechnology, Dallas, Texas) at various concentrations for 1, 2 and 3 days. The viability of DMSO-treated cells (negative control: NC) was regarded as 100%. Values of the 50% inhibitory concentration (IC_50_) were determined using sigmoidal dose-response (variable slope) statistics and normalized in GraphPad Prism. The cells were treated with an IC_50_ dose of the NPM1 inhibitor for further studies. The relative cell viability (%) was calculated using the equation OD^T^/OD^C^ × 100%, where OD^T^ represents the absorbance of the treatment group and OD^C^ represents the absorbance of the control group, as reported previously [33].

### Cell proliferation assay

The effects on cell proliferation were confirmed using the Roche Colorimetric Assay kit 1 (BrdU labeling and detection kit III; Roche Diagnostics GmbH, Germany) according to the manufacturer’s instructions. The absorbance of the samples against a background control was measured using a Microplate Reader (Molecular Devices, Sunnyvale, CA) at a wavelength of 575 nm for BrdU.

### Western blot analysis

Cells were harvested after drug treatment. After isolation of total proteins, the protein concentration was determined. Western blot analysis was performed as described previously [32]. Anti-NPM1 (1:1000; Abcam) and anti-β-actin (1:5000; Sigma-Aldrich, St. Louis, MO) antibodies were used. For native PAGE, cell lysate was not heat denatured, and a Native PAGE Novex Bis-Tris gel system was used (Life Technologies) according to the manufacturer’s protocol.

### NPM1 knockdown with siRNAs

NPM1 small interfering RNA (siRNA) and negative control siRNA were purchased from Bioneer (Daejeon, South Korea). The sequences of the NPM1 siRNAs were as follows: NPM1 siRNA-1 5′-GAAGCAGAGGCAAUGAAUUACGA-3′ (sense), 5′-CUCCGUAAUUCAUUGCCUCUGCUUCAA-3′ (antisense); NPM1 siRNA-2 5′-AGGUGGUAGCAAGGUUCCA-3′ (sense), 5′-UGGAACCUUGCACCACCU-3′ (antisense); NPM1 siRNA-3 5′-GAAAAUGAGCACCAGUUAU-3′ (sense), 3′-AUAACUGGUGCUCAUUUUC-3′ (antisense). siRNA-mediated inhibition of NPM1 expression was performed using Lipofectamine RNAiMax (Invitrogen) according to the manufacturer’s instructions. Knockdown efficiency was confirmed by RT-qPCR and Western blots 48 h after transfection with NPM1 siRNA and NC siRNA.

### Real-time quantitative reverse transcription polymerase chain reaction (RT-qPCR)

Total RNA was extracted from transfected cells using a RNeasy Plus Kit (Qiagen, Hilden, Germany). The real-time RT-qPCR analysis of mRNAs was performed using the TaqMan gene expression assay kit (Life Technologies) on an ABI 7000 system (Applied Biosystems, Foster City, CA) under the conditions specified in the ABI TaqMan assay protocol. TaqMan probes for NPM1 and glyceraldehyde 3-phosphate dehydrogenase (GAPDH) were used. All reactions were repeated in triplicate, and the comparative threshold cycle (ΔCt) method was used to calculate the relative gene expression. The results were normalized to GAPDH and are presented relative to the negative control.

### Cell cycle analysis

Cells were plated on 100-mm plates and treated with IC_50_ values of the indicated chemicals. After NPM1 inhibitor treatment at the given concentrations, cells were fixed using ice-cold 70% ethanol, washed with 1x PBS and then suspended in propidium iodide (10 μg/ml) and ribonuclease A (0.1%). Cells were incubated for 30 min in the dark at room temperature. Propidium fluorescence was quantified after laser excitation of the fluorescent dye by FACS (BD Biosciences, San Jose, CA) with a cell count of 10,000 cells per sample. Finally, the DNA content of the cells in different phases of the cell cycle was determined using CellQuest Software (BD Biosciences, San Jose, CA).

### Data availability statement

WES and RNA-seq data are available at this link: *https://www.ncbi.nlm.nih.gov/Traces/study/?acc=SRP159412**.*

## Results

### Biallelic inactivation of SMARCB1 is the only recurrent genomic alteration in AT/RTs

**Fig. 1:**
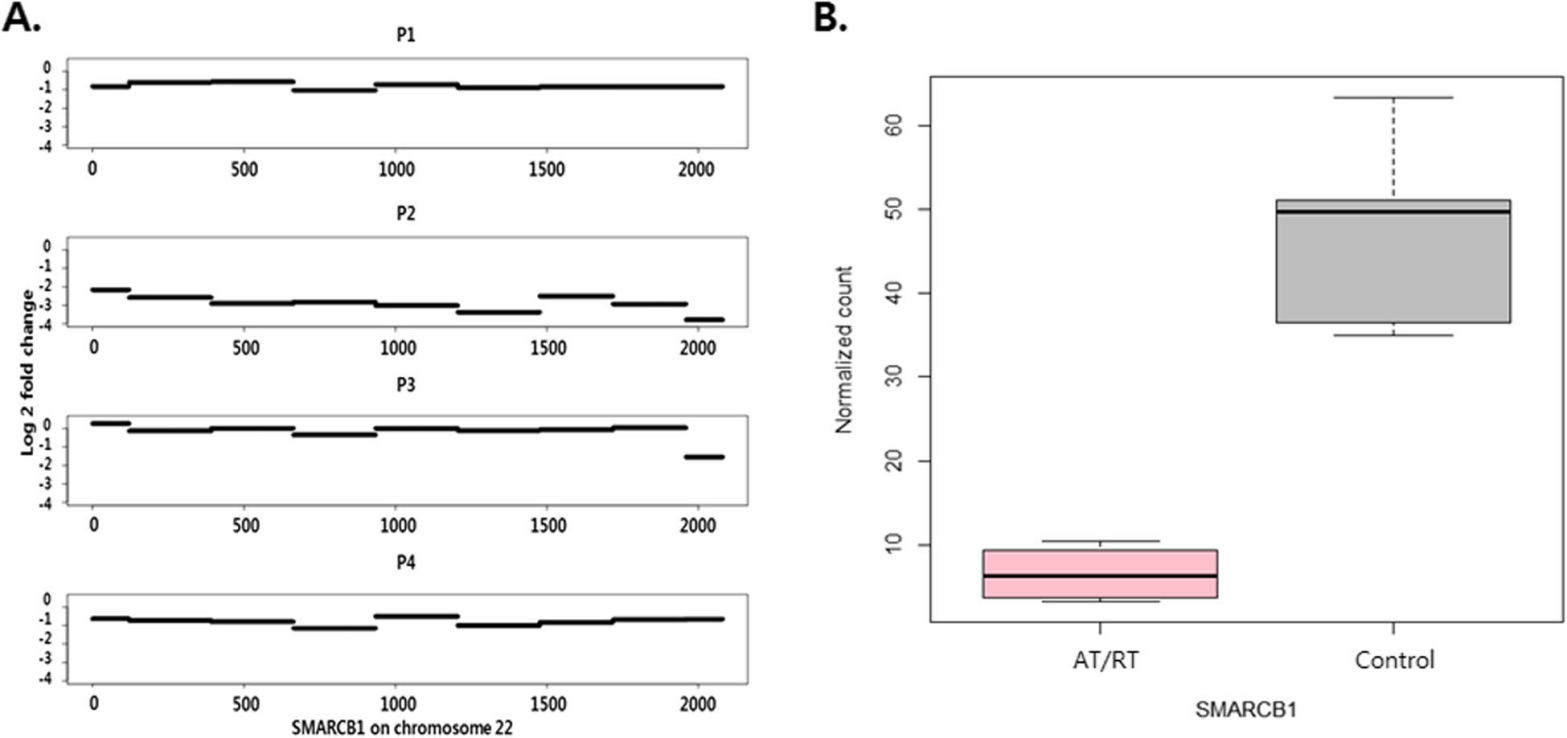
Biallelic inactivation and expression changes of SMARCB1. **a** Copy number changes in SMARCB1 exons on chromosome 22 of 4 AT/RT samples in whole exome capture sequencing. All 4 tumor samples showed loss of heterozygosity (heterozygous in the normal tissue but homozygous in the tumor tissue) based on variant allele fraction values. **b** Expression differences between the AT/RT samples and the control samples analyzed in whole transcriptome sequencing (*p*-value = 2.69E-05). Normalized reads per kilobase per million mapped reads (RPKM) values revealed a significant decrease in AT/RT samples compared with normal brain samples

Analysis of WES, SNP arrays, and aCGH revealed extremely low mutation frequencies overall in AT/RT samples, which harbored a total of only 23 somatic mutations in all patients (Additional file 1: Table S4). Among these few genomic alterations, biallelic inactivation of *SMARCB1* was the only consistent somatic alteration in the samples (Fig. 1). Various mechanisms of *SMARCB1* inactivation, such as homozygous deletion, heterozygous deletion with or without somatic mutation, and copy-neutral loss of heterozygosity (LOH), were observed (Table 1). Consistent with these genetic alterations, the expression levels of *SMARCB1* analyzed by RNA-Seq were significantly downregulated in all cases compared with controls (Fig. 1).

**Table 1:** Genetic alterations of *SMARCB1* in the AT/RT samples

Patient	Copy number alteration	Somatic mutation (type)	^a^Relative expression level
P1	Heterozygous deletion	c.811G > T (stop codon, homozygote)	−3.76
P2	Homozygous deletion	Not available	−3.42
P3	Copy neutral	c.118C > T (stop codon, homozygote)	−2.22
P4	Heterozygous deletion	c.93 + 2 T > C (splice donor, heterozygote)	−2.51

^a^log2-fold change of RPKM (sample to control)

### Inactivation of SMARCB1 results in remarkably altered expression of known cancer-associated genes

**Fig. 2:**
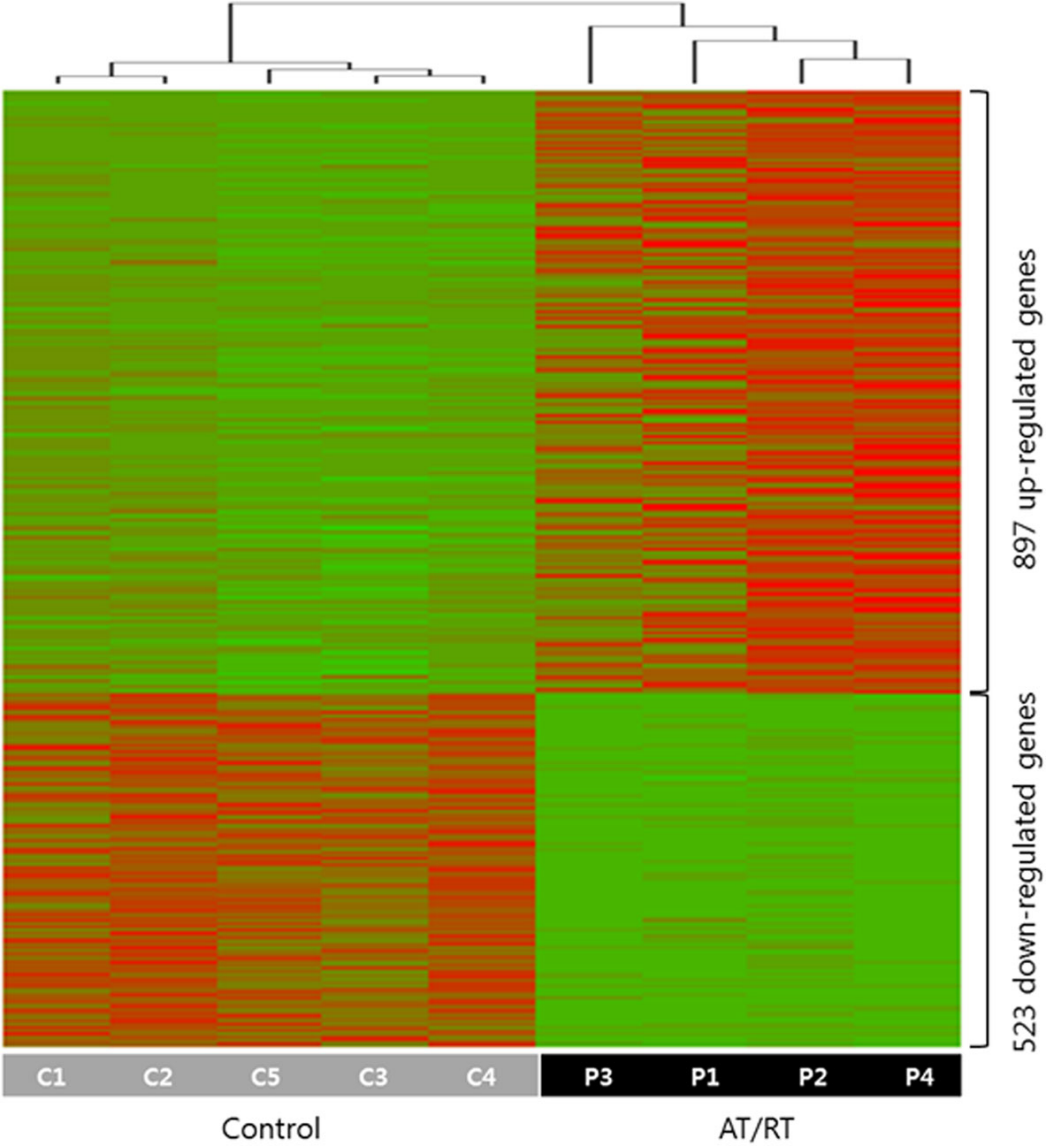
Heatmap showing differentially expressed genes (DEGs) in the AT/RT samples compared with the control samples. A total of 897 significantly upregulated genes (log2(FC) > 1, *p* < 0.05, negative binomial test) and 523 significantly downregulated genes (log2(FC) < − 1, *p* < 0.001, negative binomial test) in the AT/RT samples are displayed. The details of the genes are listed in Additional file 1: Table S5 and S6

To screen the common transcriptional cascade genes affected by *SMARCB1* mutation, we investigated DEGs between AT/RT samples and controls using the SAMSeq method in the SAMR package (R library) with the RNA-Seq data. Overall, changes in gene expression were relatively modest, and most genes had similar expression levels (RPKM fold change (FC) between 0.5 and 2) in AT/RT and control samples. However, a subset of genes displayed remarkably altered expression (Fig. 2), with 897 significantly upregulated genes (Additional file 1: Table S5; log2(FC) > 1, *p* < 0.05, negative binomial test) and 523 significantly downregulated genes (Additional file 1: Table S6; log2(FC) < − 1, *p* < 0.001, negative binomial test) in the AT/RT samples.

**Fig. 3:**
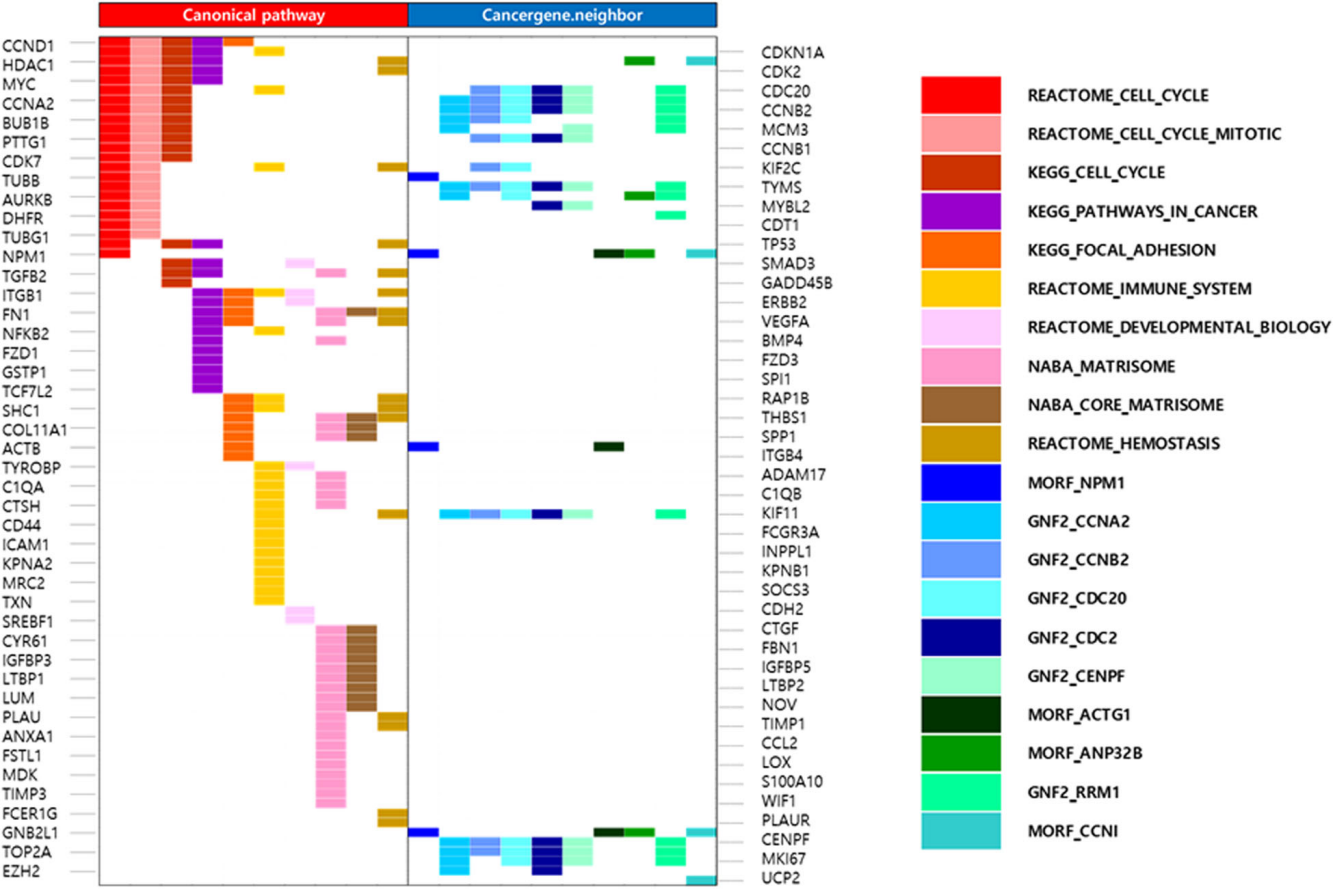
Gene sets significantly enriched by differentially expressed genes in the AT/RT samples after gene set enrichment analysis (GSEA). Ten gene sets each were significantly enriched within the ‘Canonical pathway’ and ‘Cancer gene neighbor’ categories. Genes that are associated with cancer (based on the Genetic Association Database (GAD, https://geneticassociationdb.nih.gov/)) are mapped with gene sets by color code. Among the 261 genes annotated in GSEA, a total of 88 genes were also commonly annotated to the cancer class of the GAD. NPM1 was the most significant gene that was upregulated in AT/RTs and was found to be associated with the cell cycle in the canonical pathway

Using these DEGs, we performed GSEA to identify the key oncological effectors of AT/RTs. Ten gene sets each were significantly enriched within the ‘Canonical pathway’ and ‘Cancer gene neighbor’ categories (Fig. 3 and Additional file 1: Table S7). The list of genes with their gene sets of enrichment are summarized by an overlap matrix (Additional file 1: Table S8). Among the 261 genes annotated in GSEA, a total of 88 genes were also commonly annotated to the cancer class of the GAD, and *NPM1* was the most significant gene that was upregulated in AT/RTs (Additional file 1: Table S9). Relatively elevated NPM1 mRNA expression in AT/RT was also confirmed by qPCR compared with medulloblastomas and normal brain tissues, including the cerebellum (Additional file 2: Figure S2).

### NPM1 is a potential therapeutic target in AT/RT

**Fig. 4:**
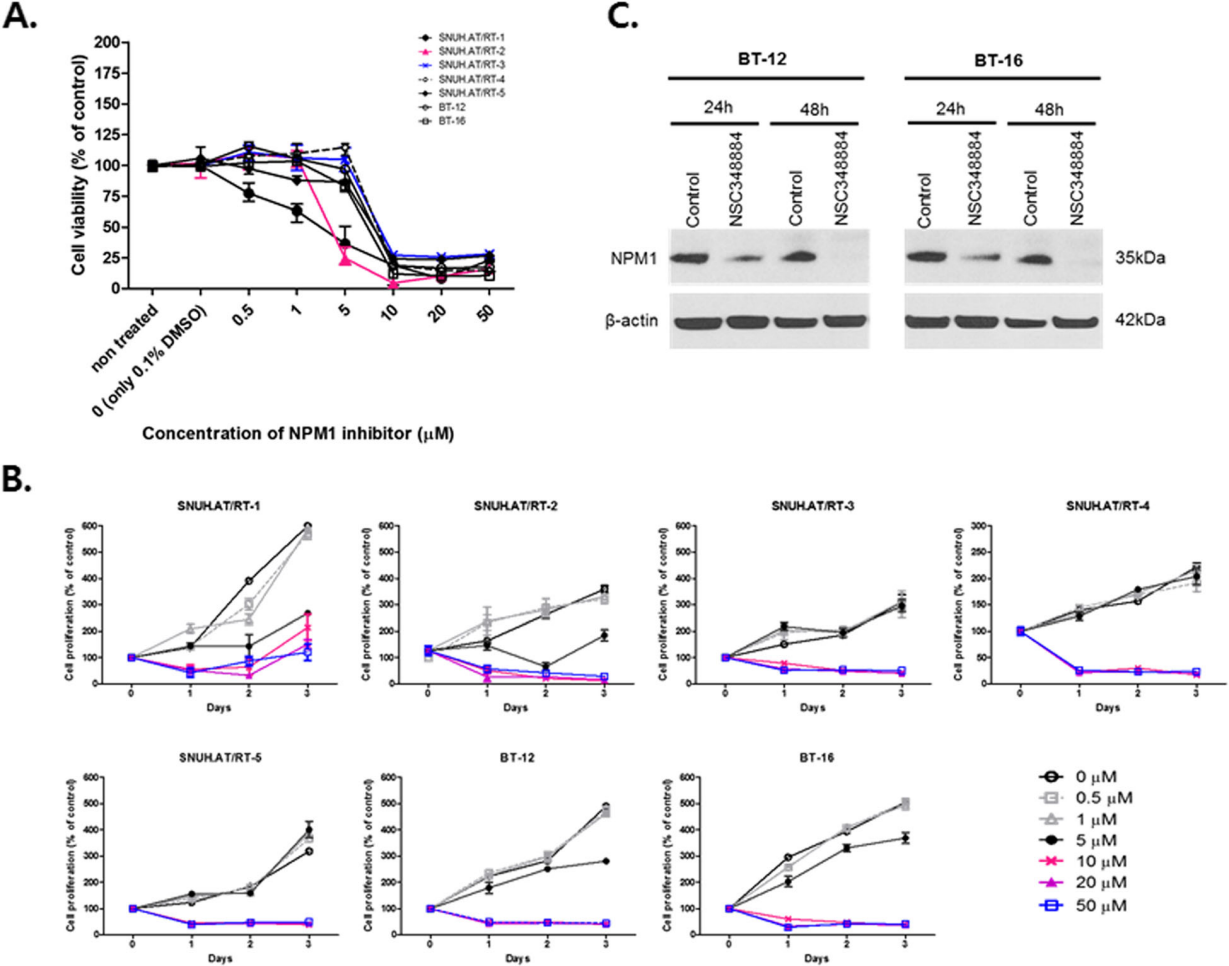
The effects of NSC348884 on multiple AT/RT cell lines. **a** A significant decrease in cell viability was observed at 48 h in all 7 AT/RT cell lines tested. The half maximal inhibitory concentration (IC_50_) ranges from 4.8 to 9.1 μM. **b** In the BrdU labeling assay, cell proliferation was also suppressed by NSC348884 at 10 μM in 72 h. **c** Western blot shows that NPM1 protein expression is effectively suppressed at IC_50_ doses of NSC348884 for each cell line. Cropped gel images are displayed for clarification

To verify whether the inactivation of NPM1 has a potential therapeutic effect in AT/RTs, in vitro experiments using NSC348884, a small molecule inhibitor that could disrupt NPM1 oligomerization, were performed. Cell viability and proliferation were examined in 7 AT/RT cell lines after NPM1 inhibition. Anti-NPM1 treatment effectively suppressed cell viability in all 7 tested cell lines (Fig. 4a). The half maximal inhibitory concentration (IC_50_) ranged from 4.8 to 9.1 μM: IC_50_ values in each cell line were 6.046 ± 0.374 μM (SNUH.AT/RT-1), 5.047 ± 0.257 μM (SNHU.AT/RT-2), 7.637 ± 0.050 μM (SNUH.AT/RT-3), 7.093 ± 0.373 μM (SNUH.AT/RT-4), 9.143 ± 0.1224 (SNUH.AT/RT-5), 4.822 ± 0.754 μM (BT-12), and 6.076 ± 0.185 μM (BT-16). In the BrdU labeling assay, cell proliferation was also suppressed by NSC348884 at 10 μM in 72 h (Fig. 4b). Western blot analysis showed that NPM1 protein expression was suppressed at IC_50_ doses of NSC348884 for each cell line (Fig. 4c). Knockdown of NPM1 with siRNAs reduced the viability of multiple AT/RT cell lines, replicating the effect of the NPM1 inhibitor (Additional file 2: Figure S3). These results suggest that transient depletion of NPM1 may affect the survival rate of AT/RT cells. Taken together, these results indicate that NPM1 is a potential target for the anticancer treatment of AT/RTs.

### Mechanism of the anticancer effect of NPM1 inhibition in AT/RTs

**Fig. 5:**
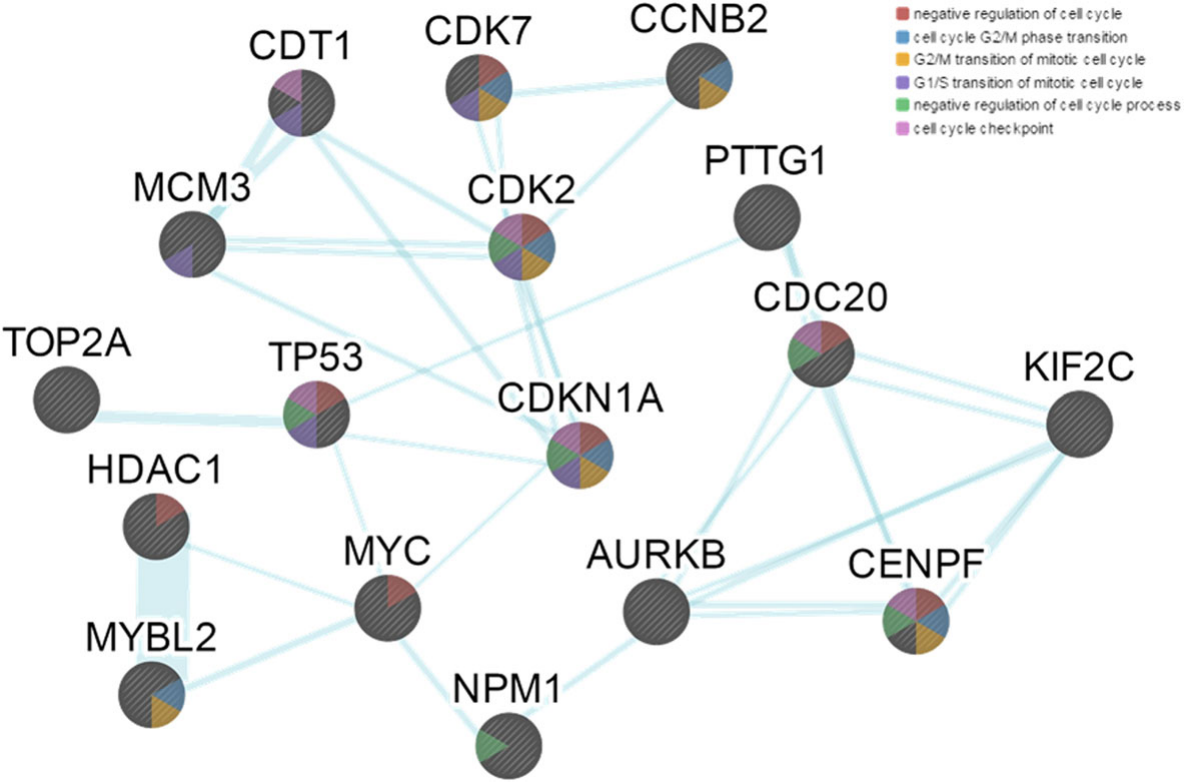
The results of the pathway analysis using genes that were closely related to NPM1 from the selected cancer-associated genes within differentially expressed genes in the AT/RT samples. All genes were annotated in cell cycle-related pathways

For deeper insight into the anticancer mechanism of NPM1 inhibition in AT/RTs, we entered the identified 88 cancer-associated genes that were deregulated in AT/RT samples into GeneMANIA software (version 3.1.2.8) for network analysis. The network was constructed using coexpression, colocalization, genetic interaction, pathway, physical interaction, and shared protein domain relationships. Then, we selected 16 genes that were closely associated with *NPM1* (*AURKB, CCNB2, CDC20, CDK2, CDK7, CDKN1A, CDT1, CENPF, HDAC1, KIF2C, MCM3, MYBL2, MYC, PTTG1, TOP2A,* and *TP53*). Pathway analysis using these selected genes, including *NPM1,* showed that most of them mapped to cell cycle-related pathways (Fig. 5). Therefore, we predicted that the anticancer effect of NPM1 inhibition in AT/RTs is mediated through the interruption of deregulated cell cycle processes. We validated the effect of NPM1 inhibition on the cell cycle in vitro using 4 AT/RT cell lines. G1 arrest was observed after NPM1 inhibitor treatment in 3 of the 4 cell lines tested (Additional file 2: Figure S4). One cell line, BT-12, showed G2 arrest after NPM1 inhibition. In all cell lines, there was a marked increase in sub-G1 fractions, indicating that apoptosis also plays a role in addition to the cell cycle effects of the NPM1 inhibitor.

## Discussion

Genetic alterations in subunits of the SWI/SNF complex are quite common and occur in up to 20% of all human cancers [34, 35]. According to accumulating evidence, biallelic inactivation of the *SMARCB1* gene has repeatedly been found to be the almost sole driving genetic alteration in AT/RTs [5, 7, 8, 36]. A variety of mechanisms, such as deletions, mutations, and loss of heterozygosity, are responsible for *SMARCB1* inactivation in AT/RTs [37]. To further elucidate the genome biology of AT/RTs, we produced a multiplanar genome dataset for 4 AT/RT samples that consisted of WES, RNA-Seq, SNP array, and aCGH analyses.

Consistent with previous reports, the biallelic inactivation of *SMACB1* was the only recurrent mutation identified in our study. We also did not find any other driver alterations in other kinds of genomic analyses, such as RNA fusion. The virtual absence of a driver oncogene in AT/RTs has been a hurdle for researchers seeking druggable targets of the disease. Recent panoramic genomic analysis of 192 AT/RTs showed that AT/RTs can be assigned to one of three subgroups with largely homogeneous genomes: AT/RT-TYR, AT/RT-SHH and AT/RT-MYC [8]. Although few differences were found between subgroups at the genetic level, the subgroups were classified by the enrichment of transcription factors and their regulatory circuits, which may be targets for therapy [8]. Moreover, analysis of genome-wide methylation patterns revealed substantial DNA hypermethylation among the AT/RT-TYR and AT/RT-SHH subtypes but not the AT/RT-MYC subgroup, which might guide the optimal treatment of patients with AT/RTs [8]. As previously reported [8], overexpression of EZH2, a component of the PRC2 complex, was observed in all AT/RTs regardless of subgroup in this study (mean log2(FC) = 2.23, *p* = 0.03, Additional file 1: Table S5). Based on the relative expression ratio of subgroup-specific signature genes compared with EZH2 in individual patient samples, the patient samples in this study could be classified into the previously proposed subgroups: AT/RT-TYR for P2 and P4, AT/RT-SHH for P1, and AT/RT-MYC for P3 (Additional file 2: Figure S5). [8] If we apply the other molecular subgroup classification proposed by Torchia et al. to the present samples, P1 was close to group 1, while the others were compatible with group 2 (Additional file 2: Figure S6) [11]. Efforts have been focusing on epigenetic changes in AT/RTs and rhabdoid tumors since it was discovered that SWI/SNF acts antagonistically toward PRC2 [38] and showed impacts on the further subclassification of AT/RTs [10]. Inhibition of EZH2, a component of PRC2, dramatically suppressed AT/RT cells in vitro and in vivo [18, 21]. However, their clinical application for anticancer therapy has yet to be studied.

Among the approaches targeting various kinds of genetic changes, cell cycle regulators are potential therapeutic targets of interest [37]. A previous study found that SMARCB1-deficient mouse embryonic fibroblasts showed a significant increase in apoptosis and cell cycle arrest with upregulation of TP53 and CDKN1A [28]. Pan-CDK inhibitors, such as flavopiridol, affected cyclinD1 and inhibited rhabdoid tumor cell growth [39]. A clinical trial of the CDK4/6 inhibitor ribociclib for patients with rhabdoid tumors and other solid cancers is currently under way [37]. In this study, we identified NPM1 as a potential therapeutic target that was universally overexpressed in AT/RT samples and showed a significant anticancer effect in vitro. In addition, the mechanism of its anticancer effect is predicted to be related to dysregulation of the cell cycle. Increasing knowledge of NPM1 has revealed its multifunctional role in cell biology, including proliferation and growth control [40]. Scanning data from The Cancer Genome Atlas (TCGA, https://tcga-data.nci.nih.gov/tcga/) revealed that alterations of NPM1 in the genome are observed in diverse types of cancers, among which acute myeloid leukemia is the most frequently affected (Additional file 2: Figure S7). Interestingly, immunohistological analyses showed universal alteration of NPM1 in human malignant rhabdoid tumors. Venneti et al. reported that NPM1/phosphorylated NPM1 were immunohistologically positive in 100%/88% of 25 AT/RTs and in 100%/100% of 11 non-CNS malignant rhabdoid tumors [41]. Together with the results of p53 and MDM2 analysis, these findings indicated the involvement of the p16^INK4A^ and p14^ARF^ tumor suppressor pathways, which resulted in cell cycle deregulation in malignant rhabdoid tumors [41]. We also showed that many cell cycle-related gene expression levels are altered in AT/RTs (Fig. 3). Moreover, other evidence has shown that SMARCB1 expression is related to cell cycle regulation. Restoration of SMARCB1 induced cellular senescence in rhabdoid cancer cell lines [42, 43]. Betz et al. showed that re-expression of SMARCB1 in pediatric tumor cells led to G1 arrest [44]. Therefore, cell cycle deregulation is one of the tumorigenesis mechanisms caused by SMARCB1 inactivation involving NPM1. Reported evidence has proposed that *NPM1* is a transcriptional target of MYC [45, 46]. As observed in our study, downregulation of NPM1 delays cell-cycle progression and the entry of cells into mitosis [47]. However, the detailed mechanism of NPM1 as a proliferation enhancer in cancer cells should be evaluated in future studies; several hypotheses have been proposed, including that NPM1 is a putative stimulatory factor for DNA polymerase-α (DNA Polα) or that NPM1 interacts with and inhibits p53 in response to apoptotic stimuli [40]. Growing evidence has shown overexpression of NPM1 in various cancers and its correlation with poor prognosis [48–55].

In our study, NSC348884 induced G1 arrest in several AT/RT cell lines (except for BT-12) (Additional file 2: Figure S4). The inconsistent effects on different cell lines may be attributed to subgroup heterogeneity of AT/RTs. Despite the stable genomic status, transcriptome and methylome profiling demonstrated that AT/RTs are a heterogeneous group of tumors [8, 11]. This subgroup difference can lead to inhomogeneous responses to target drugs. Protein expression of BT-12 showed the characteristics of AT/RT-TYR, whereas BT-16 was close to AT/RT-SHH (Additional file 2: Figure S8). Interestingly, BT-12 was also the only cell line in which NPM1 siRNA exerted no effect on cell viability (Additional file 2: Figure S3). The subgroup difference in response to drugs is of interest because of the potential of tailored therapy to each tumor subgroup. However, little is known about the therapeutic targets of each AT/RT subgroup. Drug screening tests on primary-cultured AT/RT cells using different types of target agents would enhance the understanding of AT/RT subgroups and prove useful in future clinical trials.

Mounting evidence has shown that NPM1 is a promising therapeutic target for the treatment of solid cancers. In addition, we propose NPM1 as a druggable target for AT/RTs. The NPM1 inhibitor used in the present study is NSC348884, which is the first small molecule developed that specifically interacts with NPM1 [56]. NSC348884 induces the loss of oligomerization of NPM1 by targeting its N-terminal domain, which causes functional impairment [58]. The apoptotic effect and cell growth inhibition caused by NSC348884 have been demonstrated previously in diverse cancer cell lines [57–60]. Many other molecules targeting NPM1 have also been developed for cancer treatment [58]. Although testing multiple compounds with diverse strategies for targeting NPM1 is needed to establish a more sophisticated protocol, NPM1 may be a promising therapeutic target for AT/RT treatment.

## Conclusion

In this study, we showed that the transcriptional profile of AT/RT is highly deregulated despite the exceptional stability of the genome. Therapeutic targets of AT/RTs can be identified among the deregulated genes. We demonstrated that NPM1, a cell cycle promoter, is the most highly upregulated gene in AT/RTs. Pharmacological inhibition of NPM1 effectively abrogated the viability of AT/RT cell lines through cell cycle arrest at the G1 phase. Therefore, we propose that NPM1 is a novel therapeutic target for AT/RTs.

## Additional files

Additional file 1: Supplementary figures. (XLSX 672 kb)

Additional file 2: Supplementary tables. (DOCX 1809 kb)

## Data Availability

WES and RNA-seq data are available at this link: *https://www.ncbi.nlm.nih.gov/Traces/study/?acc=SRP159412**.*

## Abbreviations

aCGH: Array comparative genomic hybridization
AT/RTs: Atypical teratoid/rhabdoid tumors
BAF: B allele frequency
CAN: Copy number alteration
DEG: Differentially expressed gene
FBS: Fetal bovine serum
FC: Fold change
GAD: Genetic Association Database
GAPDH: Glyceraldehyde 3-phosphate dehydrogenase
GSEA: Gene set enrichment analysis
LOH: Loss of heterozygosity
MEFs: Mouse embryonic fibroblasts
NPM1: Nucleophosmin
PRC2: Polycomb-repressive complex 2
PSCBS: Parent specific circular binary segmentation
RIN: RNA Integrity Number
RNA-Seq: Whole transcriptome sequencing
SNP: Single nucleotide polymorphism
SWI/SNF: Switch/sucrose non-fermentable chromatin remodeling
VAF: Variant allele fraction
WES: Whole exome sequencing

## References

[1] von Hoff K, Hinkes B, Dannenmann-Stern E, von Bueren AO, Warmuth-Metz M, Soerensen N, Emser A, Zwiener I, Schlegel PG, Kuehl J (2011). Frequency, risk-factors and survival of children with atypical teratoid rhabdoid tumors (AT/RT) of the CNS diagnosed between 1988 and 2004, and registered to the German HIT database. Pediatr Blood Cancer.

[2] Lee JY, Kim IK, Phi JH, Wang KC, Cho BK, Park SH, Ahn HS, Kim IH, Kim SK (2012). Atypical teratoid/rhabdoid tumors: the need for more active therapeutic measures in younger patients. J Neuro-Oncol.

[3] Chi SN, Zimmerman MA, Yao X, Cohen KJ, Burger P, Biegel JA, Rorke-Adams LB, Fisher MJ, Janss A, Mazewski C (2009). Intensive multimodality treatment for children with newly diagnosed CNS atypical teratoid rhabdoid tumor. J Clin Oncol.

[4] Lafay-Cousin L, Hawkins C, Carret AS, Johnston D, Zelcer S, Wilson B, Jabado N, Scheinemann K, Eisenstat D, Fryer C (2012). Central nervous system atypical teratoid rhabdoid tumours: the Canadian Paediatric brain tumour consortium experience. Eur J Cancer.

[5] Hasselblatt M, Isken S, Linge A, Eikmeier K, Jeibmann A, Oyen F, Nagel I, Richter J, Bartelheim K, Kordes U (2013). High-resolution genomic analysis suggests the absence of recurrent genomic alterations other than SMARCB1 aberrations in atypical teratoid/rhabdoid tumors. Genes Chromosomes Cancer.

[6] Wilson BG, Roberts CW (2011). SWI/SNF nucleosome remodellers and cancer. Nature Rev Cancer.

[7] Hoell JI, Gombert M, Bartenhagen C, Ginzel S, Husemann P, Felsberg J, Reifenberger G, Eggert A, Dugas M, Schonberger S (2013). Whole-genome paired-end analysis confirms remarkable genomic stability of atypical teratoid/rhabdoid tumors. Genes Chromosomes Cancer.

[8] Johann PD, Erkek S, Zapatka M, Kerl K, Buchhalter I, Hovestadt V, Jones DT, Sturm D, Hermann C, Segura Wang M (2016). Atypical Teratoid/Rhabdoid tumors are comprised of three epigenetic subgroups with distinct enhancer landscapes. Cancer Cell.

[9] Lawrence MS, Stojanov P, Polak P, Kryukov GV, Cibulskis K, Sivachenko A, Carter SL, Stewart C, Mermel CH, Roberts SA (2013). Mutational heterogeneity in cancer and the search for new cancer-associated genes. Nature.

[10] Torchia J, Golbourn B, Feng S, Ho KC, Sin-Chan P, Vasiljevic A, Norman JD, Guilhamon P, Garzia L, Agamez NR (2016). Integrated (epi)-genomic analyses identify subgroup-specific therapeutic targets in CNS Rhabdoid tumors. Cancer Cell.

[11] Torchia J, Picard D, Lafay-Cousin L, Hawkins CE, Kim SK, Letourneau L, Ra YS, Ho KC, Chan TS, Sin-Chan P (2015). Molecular subgroups of atypical teratoid rhabdoid tumours in children: an integrated genomic and clinicopathological analysis. Lancet Oncol.

[12] Birks DK, Donson AM, Patel PR, Sufit A, Algar EM, Dunham C, Kleinschmidt-DeMasters BK, Handler MH, Vibhakar R, Foreman NK (2013). Pediatric rhabdoid tumors of kidney and brain show many differences in gene expression but share dysregulation of cell cycle and epigenetic effector genes. Pediatr Blood Cancer.

[13] Guidi CJ, Sands AT, Zambrowicz BP, Turner TK, Demers DA, Webster W, Smith TW, Imbalzano AN, Jones SN (2001). Disruption of Ini1 leads to peri-implantation lethality and tumorigenesis in mice. Mol Cell Biol.

[14] Ng JM, Martinez D, Marsh ED, Zhang Z, Rappaport E, Santi M, Curran T (2015). Generation of a mouse model of atypical teratoid/rhabdoid tumor of the central nervous system through combined deletion of Snf5 and p53. Cancer Res.

[15] Kieran MW, Roberts CW, Chi SN, Ligon KL, Rich BE, Macconaill LE, Garraway LA, Biegel JA (2012). Absence of oncogenic canonical pathway mutations in aggressive pediatric rhabdoid tumors. Pediatr Blood Cancer.

[16] Lee S, Cimica V, Ramachandra N, Zagzag D, Kalpana GV (2011). Aurora a is a repressed effector target of the chromatin remodeling protein INI1/hSNF5 required for rhabdoid tumor cell survival. Cancer Res.

[17] Helming KC, Wang X, Roberts CW (2014). Vulnerabilities of mutant SWI/SNF complexes in cancer. Cancer Cell.

[18] Alimova I, Birks DK, Harris PS, Knipstein JA, Venkataraman S, Marquez VE, Foreman NK, Vibhakar R (2013). Inhibition of EZH2 suppresses self-renewal and induces radiation sensitivity in atypical rhabdoid teratoid tumor cells. Neuro-Oncology.

[19] Tsikitis M, Zhang Z, Edelman W, Zagzag D, Kalpana GV (2005). Genetic ablation of cyclin D1 abrogates genesis of rhabdoid tumors resulting from Ini1 loss. Proc Natl Acad Sci U S A.

[20] Zhang ZK, Davies KP, Allen J, Zhu L, Pestell RG, Zagzag D, Kalpana GV (2002). Cell cycle arrest and repression of cyclin D1 transcription by INI1/hSNF5. Mol Cell Biol.

[21] Knutson SK, Warholic NM, Wigle TJ, Klaus CR, Allain CJ, Raimondi A, Porter Scott M, Chesworth R, Moyer MP, Copeland RA (2013). Durable tumor regression in genetically altered malignant rhabdoid tumors by inhibition of methyltransferase EZH2. Proc Natl Acad Sci U S A.

[22] Tolstorukov MY, Sansam CG, Lu P, Koellhoffer EC, Helming KC, Alver BH, Tillman EJ, Evans JA, Wilson BG, Park PJ (2013). Swi/Snf chromatin remodeling/tumor suppressor complex establishes nucleosome occupancy at target promoters. Proc Natl Acad Sci U S A.

[23] Abecasis GR, Auton A, Brooks LD, DePristo MA, Durbin RM, Handsaker RE, Kang HM, Marth GT, McVean GA (2012). An integrated map of genetic variation from 1,092 human genomes. Nature.

[24] Cingolani P, Platts A, Wang le L, Coon M, Nguyen T, Wang L, Land SJ, Lu X, Ruden DM (2012). A program for annotating and predicting the effects of single nucleotide polymorphisms, SnpEff: SNPs in the genome of Drosophila melanogaster strain w1118; iso-2; iso-3. Fly.

[25] Li J, Lupat R, Amarasinghe KC, Thompson ER, Doyle MA, Ryland GL, Tothill RW, Halgamuge SK, Campbell IG, Gorringe KL (2012). CONTRA: copy number analysis for targeted resequencing. Bioinformatics.

[26] Trapnell C, Pachter L, Salzberg SL (2009). TopHat: discovering splice junctions with RNA-Seq. Bioinformatics.

[27] Anders S, Huber W (2010). Differential expression analysis for sequence count data. Genome Biol.

[28] Isakoff MS, Sansam CG, Tamayo P, Subramanian A, Evans JA, Fillmore CM, Wang X, Biegel JA, Pomeroy SL, Mesirov JP (2005). Inactivation of the Snf5 tumor suppressor stimulates cell cycle progression and cooperates with p53 loss in oncogenic transformation. Proc Natl Acad Sci U S A.

[29] Van Loo P, Nordgard SH, Lingjaerde OC, Russnes HG, Rye IH, Sun W, Weigman VJ, Marynen P, Zetterberg A, Naume B (2010). Allele-specific copy number analysis of tumors. Proc Natl Acad Sci U S A.

[30] Subramanian A, Tamayo P, Mootha VK, Mukherjee S, Ebert BL, Gillette MA, Paulovich A, Pomeroy SL, Golub TR, Lander ES (2005). Gene set enrichment analysis: a knowledge-based approach for interpreting genome-wide expression profiles. Proc Natl Acad Sci U S A.

[31] Warde-Farley D, Donaldson SL, Comes O, Zuberi K, Badrawi R, Chao P, Franz M, Grouios C, Kazi F, Lopes CT (2010). The GeneMANIA prediction server: biological network integration for gene prioritization and predicting gene function. Nucleic Acids Res.

[32] Choi SA, Kim SK, Lee JY, Wang KC, Lee C, Phi JH (2016). LIN28B is highly expressed in atypical teratoid/rhabdoid tumor (AT/RT) and suppressed through the restoration of SMARCB1. Cancer Cell Int.

[33] Choi SA, Choi JW, Wang KC, Phi JH, Lee JY, Park KD, Eum D, Park SH, Kim IH, Kim SK (2015). Disulfiram modulates stemness and metabolism of brain tumor initiating cells in atypical teratoid/rhabdoid tumors. Neuro-Oncology.

[34] Biegel JA, Busse TM, Weissman BE (2014). SWI/SNF chromatin remodeling complexes and cancer. Am J Med Genet C Semin Med Genet.

[35] Kadoch C, Hargreaves DC, Hodges C, Elias L, Ho L, Ranish J, Crabtree GR (2013). Proteomic and bioinformatic analysis of mammalian SWI/SNF complexes identifies extensive roles in human malignancy. Nature Genet.

[36] Lee RS, Stewart C, Carter SL, Ambrogio L, Cibulskis K, Sougnez C, Lawrence MS, Auclair D, Mora J, Golub TR (2012). A remarkably simple genome underlies highly malignant pediatric rhabdoid cancers. J Clin Invest.

[37] Fruhwald MC, Biegel JA, Bourdeaut F, Roberts CW, Chi SN (2016). Atypical teratoid/rhabdoid tumors-current concepts, advances in biology, and potential future therapies. Neuro-Oncology.

[38] Kia SK, Gorski MM, Giannakopoulos S, Verrijzer CP (2008). SWI/SNF mediates polycomb eviction and epigenetic reprogramming of the INK4b-ARF-INK4a locus. Mol Cell Biol.

[39] Smith ME, Cimica V, Chinni S, Jana S, Koba W, Yang Z, Fine E, Zagzag D, Montagna C, Kalpana GV (2011). Therapeutically targeting cyclin D1 in primary tumors arising from loss of Ini1. Proc Natl Acad Sci U S A.

[40] Grisendi S, Mecucci C, Falini B, Pandolfi PP (2006). Nucleophosmin and cancer. Nat Rev Cancer.

[41] Venneti S, Le P, Martinez D, Eaton KW, Shyam N, Jordan-Sciutto KL, Pawel B, Biegel JA, Judkins AR (2011). p16INK4A and p14ARF tumor suppressor pathways are deregulated in malignant rhabdoid tumors. J Neuropathol Exp Neurol.

[42] Chai J, Charboneau AL, Betz BL, Weissman BE (2005). Loss of the hSNF5 gene concomitantly inactivates p21CIP/WAF1 and p16INK4a activity associated with replicative senescence in A204 rhabdoid tumor cells. Cancer Res.

[43] Oruetxebarria I, Venturini F, Kekarainen T, Houweling A, Zuijderduijn LM, Mohd-Sarip A, Vries RG, Hoeben RC, Verrijzer CP (2004). P16INK4a is required for hSNF5 chromatin remodeler-induced cellular senescence in malignant rhabdoid tumor cells. J Biol Chem.

[44] Betz BL, Strobeck MW, Reisman DN, Knudsen ES, Weissman BE (2002). Re-expression of hSNF5/INI1/BAF47 in pediatric tumor cells leads to G1 arrest associated with induction of p16ink4a and activation of RB. Oncogene.

[45] Boon K, Caron HN, van Asperen R, Valentijn L, Hermus MC, van Sluis P, Roobeek I, Weis I, Voute PA, Schwab M (2001). N-myc enhances the expression of a large set of genes functioning in ribosome biogenesis and protein synthesis. EMBO J.

[46] Zeller KI, Haggerty TJ, Barrett JF, Guo Q, Wonsey DR, Dang CV (2001). Characterization of nucleophosmin (B23) as a Myc target by scanning chromatin immunoprecipitation. J Biol Chem.

[47] Jiang PS, Yung BY (1999). Down-regulation of nucleophosmin/B23 mRNA delays the entry of cells into mitosis. Biochem Biophys Res Commun.

[48] Bernard K, Litman E, Fitzpatrick JL, Shellman YG, Argast G, Polvinen K, Everett AD, Fukasawa K, Norris DA, Ahn NG (2003). Functional proteomic analysis of melanoma progression. Cancer Res.

[49] Chen J, Sun J, Yang L, Yan Y, Shi W, Shi J, Huang Q, Chen J, Lan Q (2015). Upregulation of B23 promotes tumor cell proliferation and predicts poor prognosis in glioma. Biochem Biophys Res Commun.

[50] Lin CY, Chao A, Wang TH, Lee LY, Yang LY, Tsai CL, Wang HS, Lai CH (2016). Nucleophosmin/B23 is a negative regulator of estrogen receptor alpha expression via AP2gamma in endometrial cancer cells. Oncotarget.

[51] Nozawa Y, Van Belzen N, Van der Made AC, Dinjens WN, Bosman FT (1996). Expression of nucleophosmin/B23 in normal and neoplastic colorectal mucosa. J Pathol.

[52] Shields LB, Gercel-Taylor C, Yashar CM, Wan TC, Katsanis WA, Spinnato JA, Taylor DD (1997). Induction of immune responses to ovarian tumor antigens by multiparity. J Soc Gynecol Investig.

[53] Subong EN, Shue MJ, Epstein JI, Briggman JV, Chan PK, Partin AW (1999). Monoclonal antibody to prostate cancer nuclear matrix protein (PRO:4-216) recognizes nucleophosmin/B23. Prostate.

[54] Tsui KH, Juang HH, Lee TH, Chang PL, Chen CL, Yung BY (2008). Association of nucleophosmin/B23 with bladder cancer recurrence based on immunohistochemical assessment in clinical samples. Acta Pharmacol Sin.

[55] Zhou F, Chen E, You D, Song Y, Sun Z, Yue L (2016). Both high expression of nucleophosmin/B23 and CRM1 predicts poorer prognosis in human gastric cancer. APMIS.

[56] Qi W, Shakalya K, Stejskal A, Goldman A, Beeck S, Cooke L, Mahadevan D (2008). NSC348884, a nucleophosmin inhibitor disrupts oligomer formation and induces apoptosis in human cancer cells. Oncogene.

[57] Balusu R, Fiskus W, Rao R, Chong DG, Nalluri S, Mudunuru U, Ma H, Chen L, Venkannagari S, Ha K (2011). Targeting levels or oligomerization of nucleophosmin 1 induces differentiation and loss of survival of human AML cells with mutant NPM1. Blood.

[58] Di Matteo A, Franceschini M, Chiarella S, Rocchio S, Travaglini-Allocatelli C, Federici L (2016). Molecules that target nucleophosmin for cancer treatment: an update. Oncotarget.

[59] Lindstrom MS, Zhang Y (2006). B23 and ARF: friends or foes?. Cell Biochem Biophys.

[60] Maiguel DA, Jones L, Chakravarty D, Yang C, Carrier F (2004). Nucleophosmin sets a threshold for p53 response to UV radiation. Mol Cell Biol.

