# Supplementary material for: NPM1 as a potential therapeutic target for atypical teratoid/rhabdoid tumors
Source: BMC Cancer. 2019 Aug 28;19:848. doi: 10.1186/s12885-019-6044-z (PMC6714307; doi:10.1186/s12885-019-6044-z)
Supplement: Supplementary file 2 — Supplementary tables. (DOCX 1809 kb) [file 12885_2019_6044_MOESM2_ESM.docx]

**NPM1 as a potential therapeutic target for atypical teratoid/rhabdoid tumor**

***Supplementary figures***

Ji Hoon Phi, Choong-Hyun Sun, Se-Hoon Lee, Seungmook Lee, Inho Park, Seung Ah Choi, Sung-Hye Park, Ji Yeoun Lee, Kyu-Chang Wang, Seung-Ki Kim, Hongseok Yun, and Chul-Kee Park

**Figure S1.** Genome copy number changes estimated by ASCAT.

**P2**


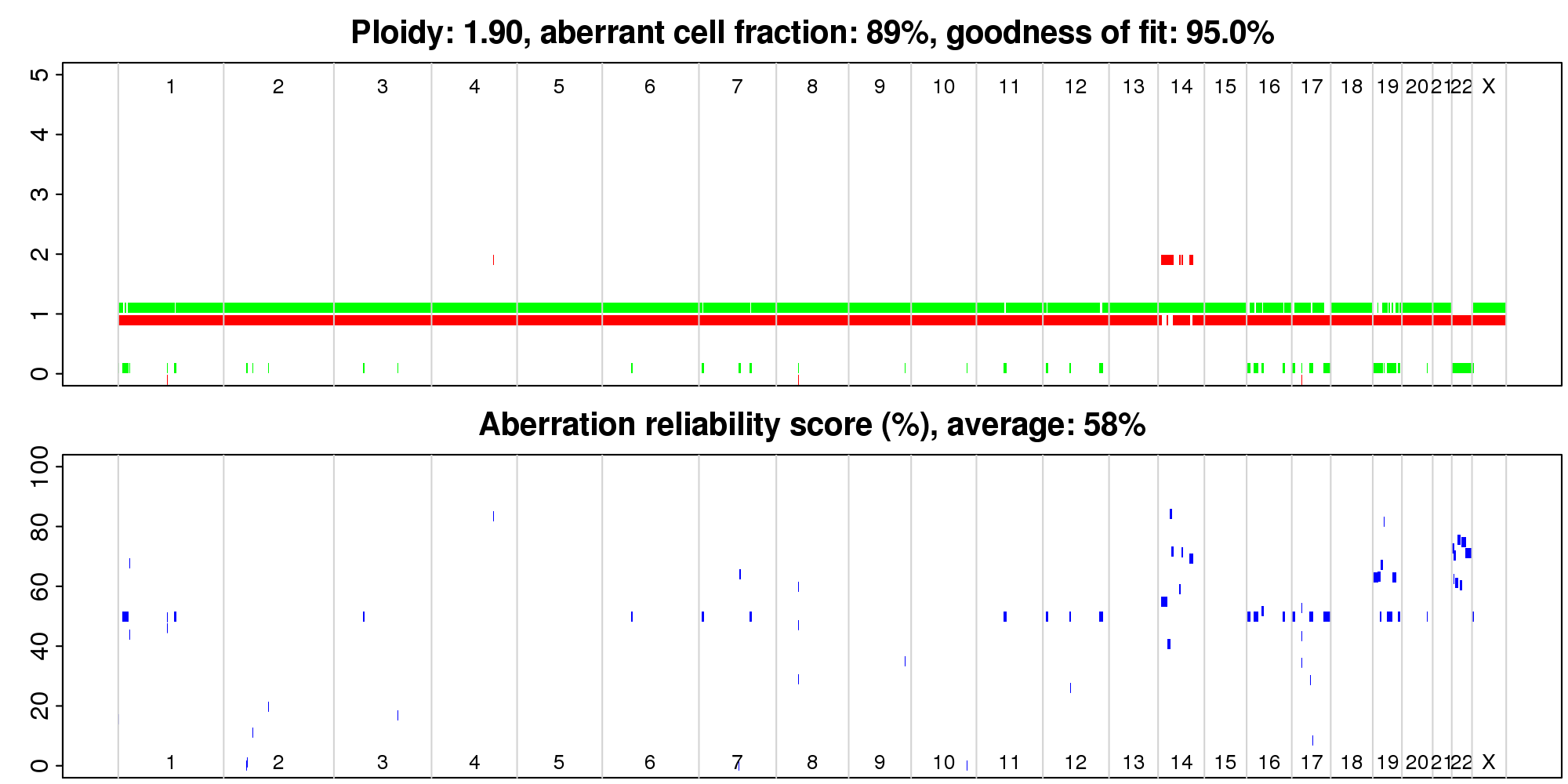

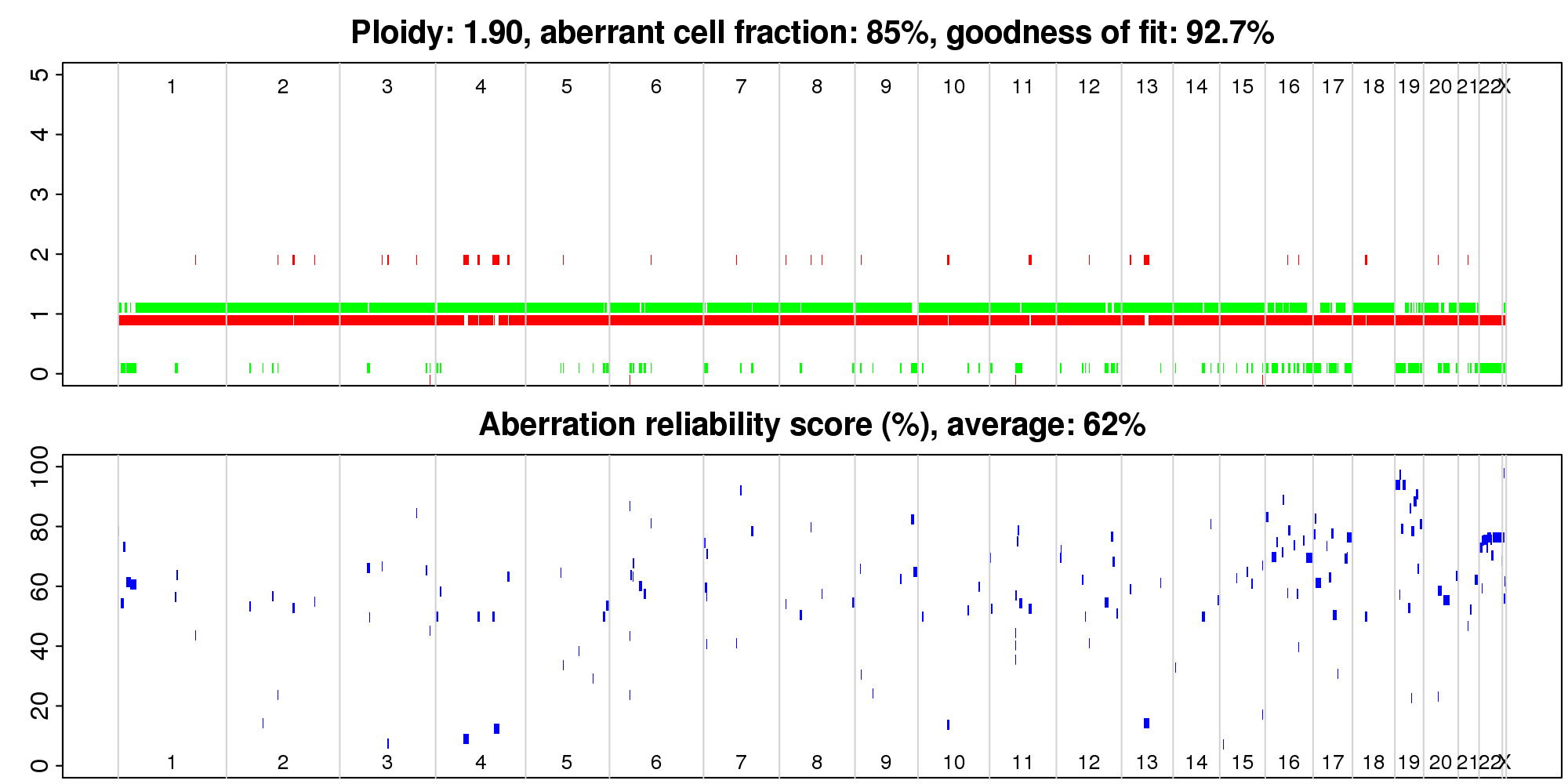


**P4**

**P1**


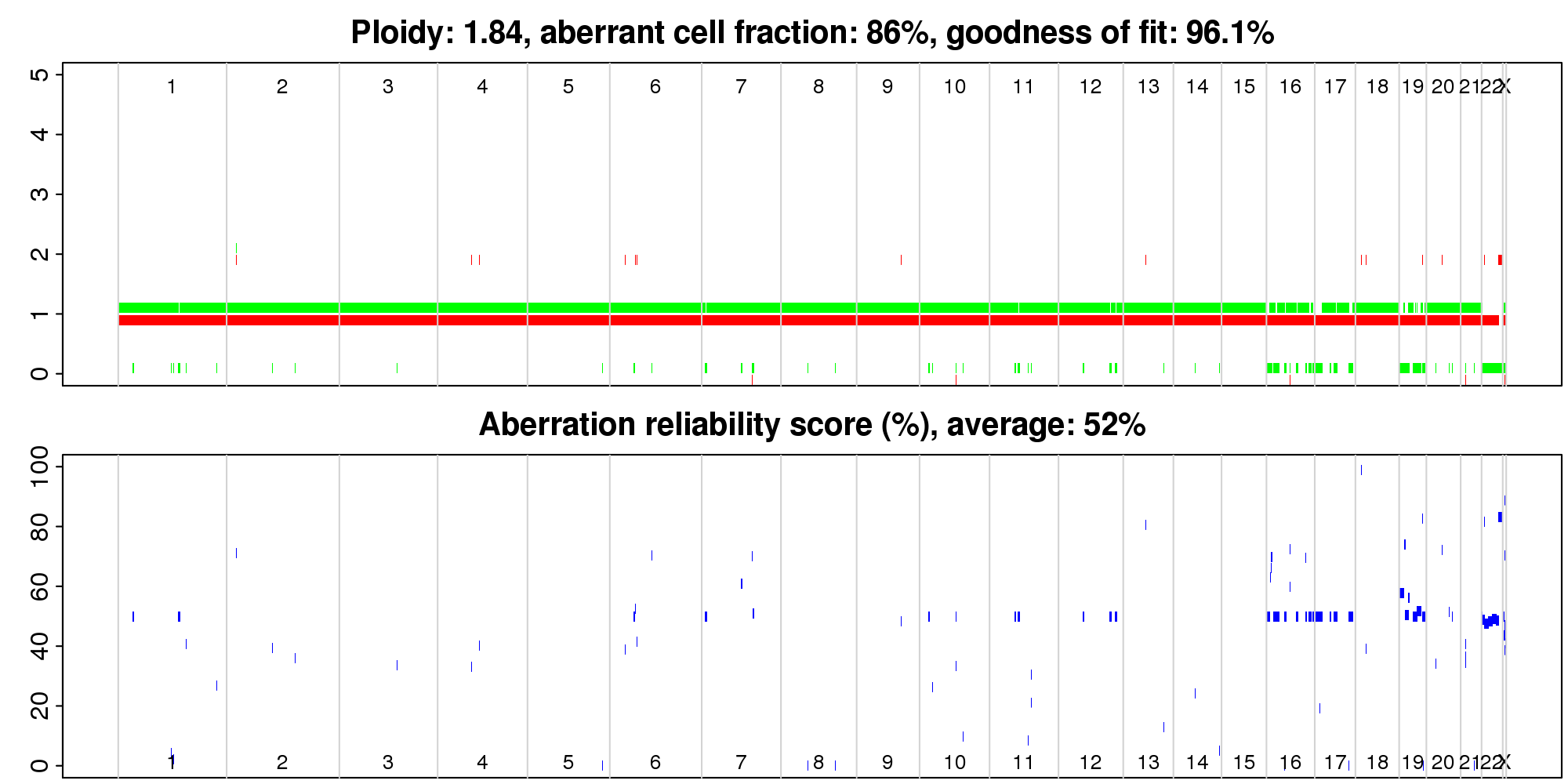


**Figure S2.** Relative mRNA expression levels of NPM1 by quantitative PCR in AT/RT, medulloblastoma, various regions of human brain and cultured human astrocytes

**Figure S3.** Transfection efficiency and cell viability in AT/RT cells 48 hours after transfection with NPM1 siRNA and NC siRNA. (A) After NPM1 knockdown, relative expression of NPM1 mRNA was decreased in AT/RT cells. (B) Representative western blot images showed that NPM1 protein expression was also effectively reduced by NPM1 siRNA transfection in all AT/RT cells. (C) Knockdown of NPM1 significantly inhibited the cell viability of AT/RT cells. *P<0.05; **P<0.01, ***P<0.001

**
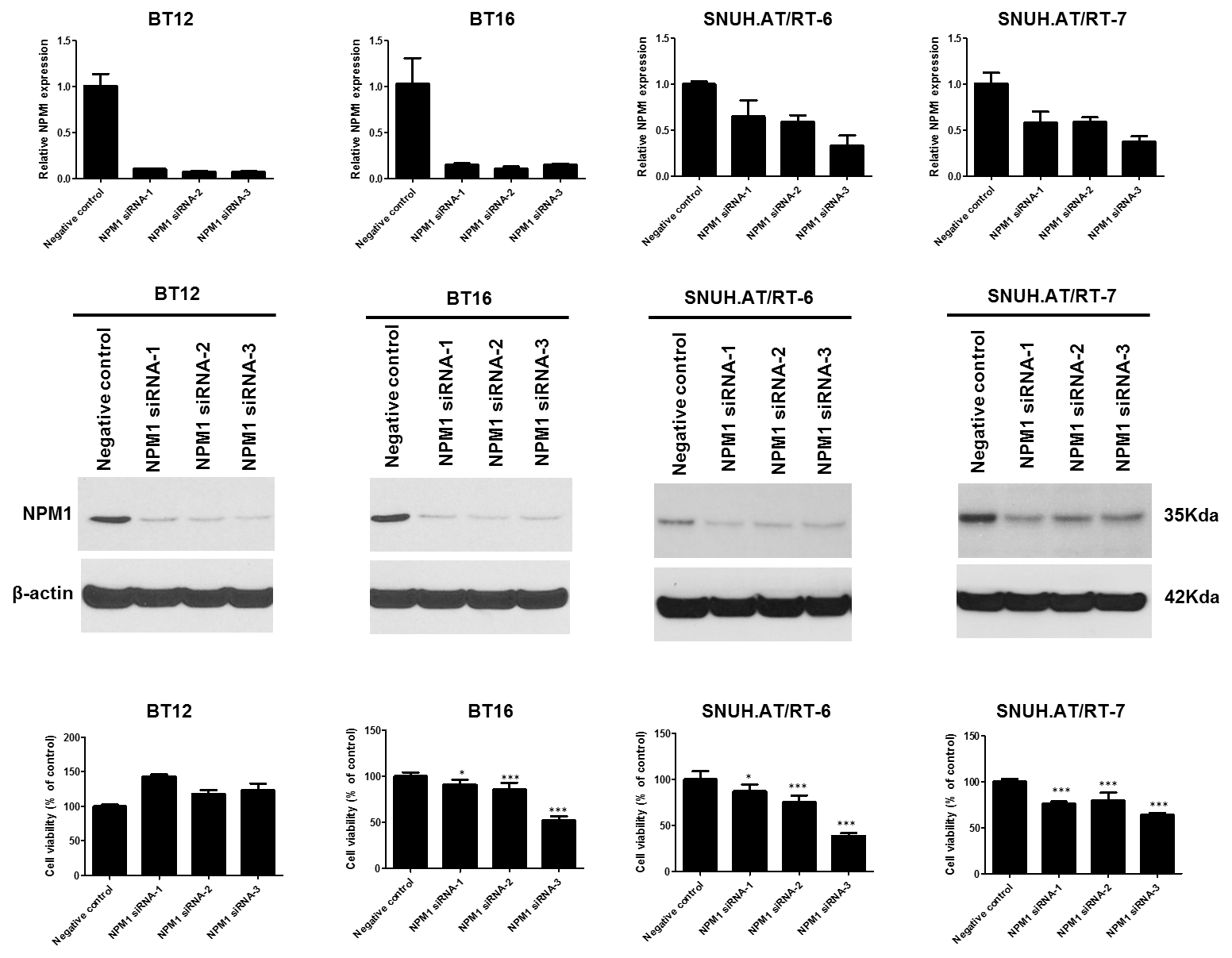
**

**Figure S4.** Flow cytometric cell cycle analysis indicates that except for BT-12 which exhibits G2 arrest, all the other cell lines show G1 arrest at 48 hours after NSC348884 treatment. In all cell lines, there was a marked increase in sub-G1 fractions indicating that apoptosis also plays a role in addition to cell cycle effects of NSC34888

**
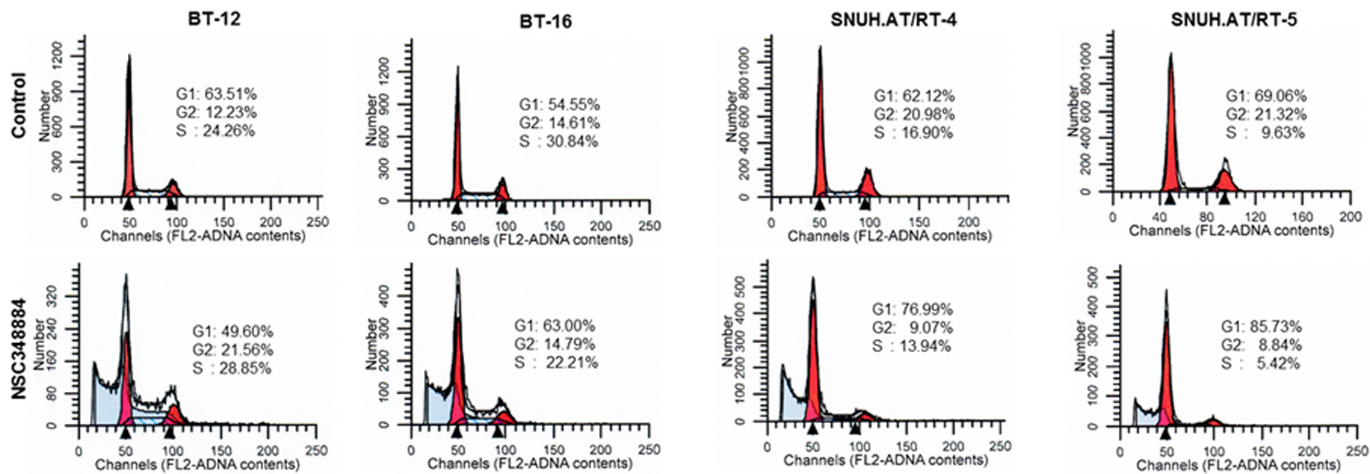
**

**Figure S5.** Relative expression ratio of previously proposed subgroup-specific signature genes (compared with individual EZH2 expression) of each patient samples used in the present study [1].


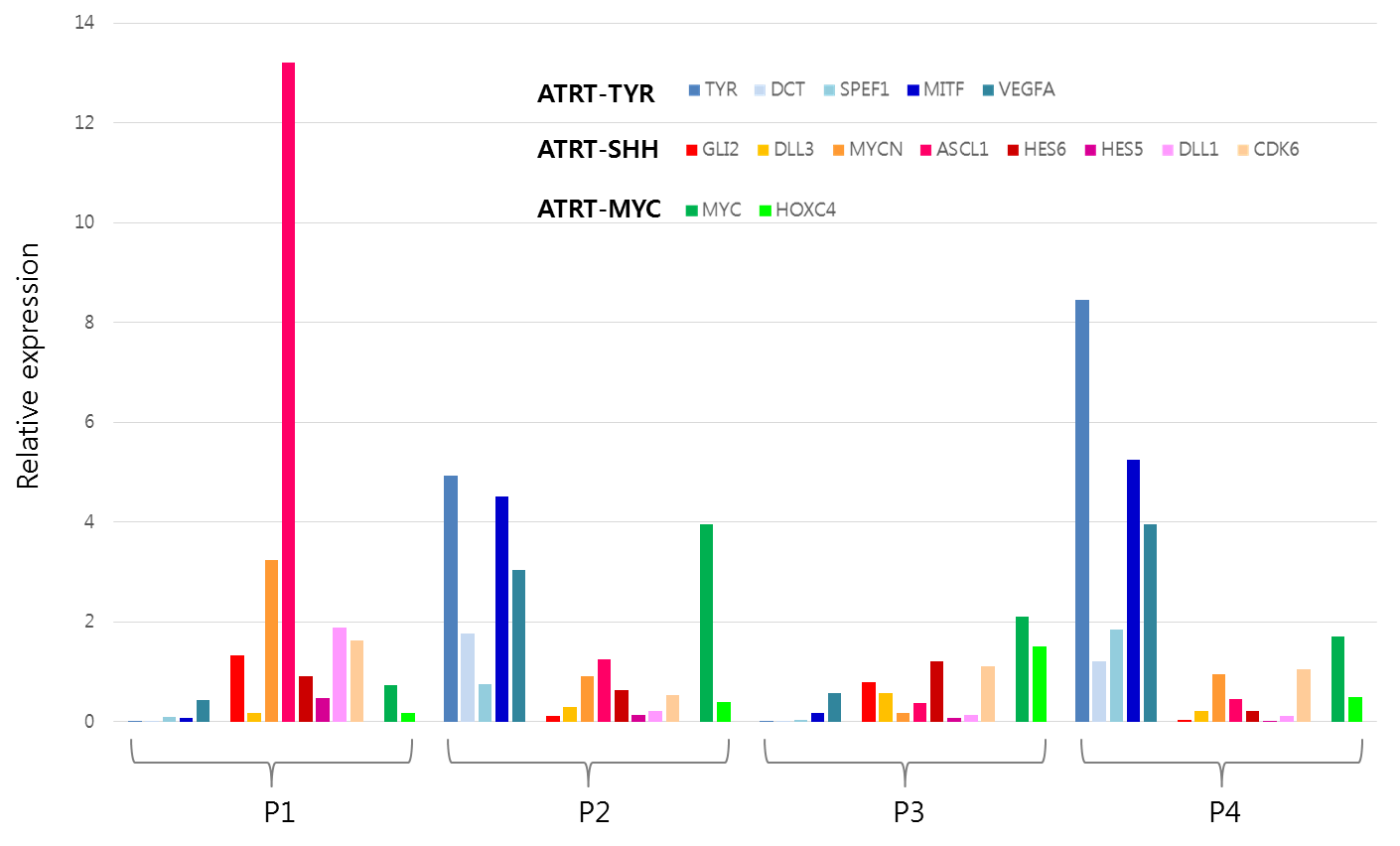


**Figure S6.** Relative expression ratio of previously proposed subgroup-specific signature genes (compared with expression in normal brain) of each patient samples used in the present study [2].


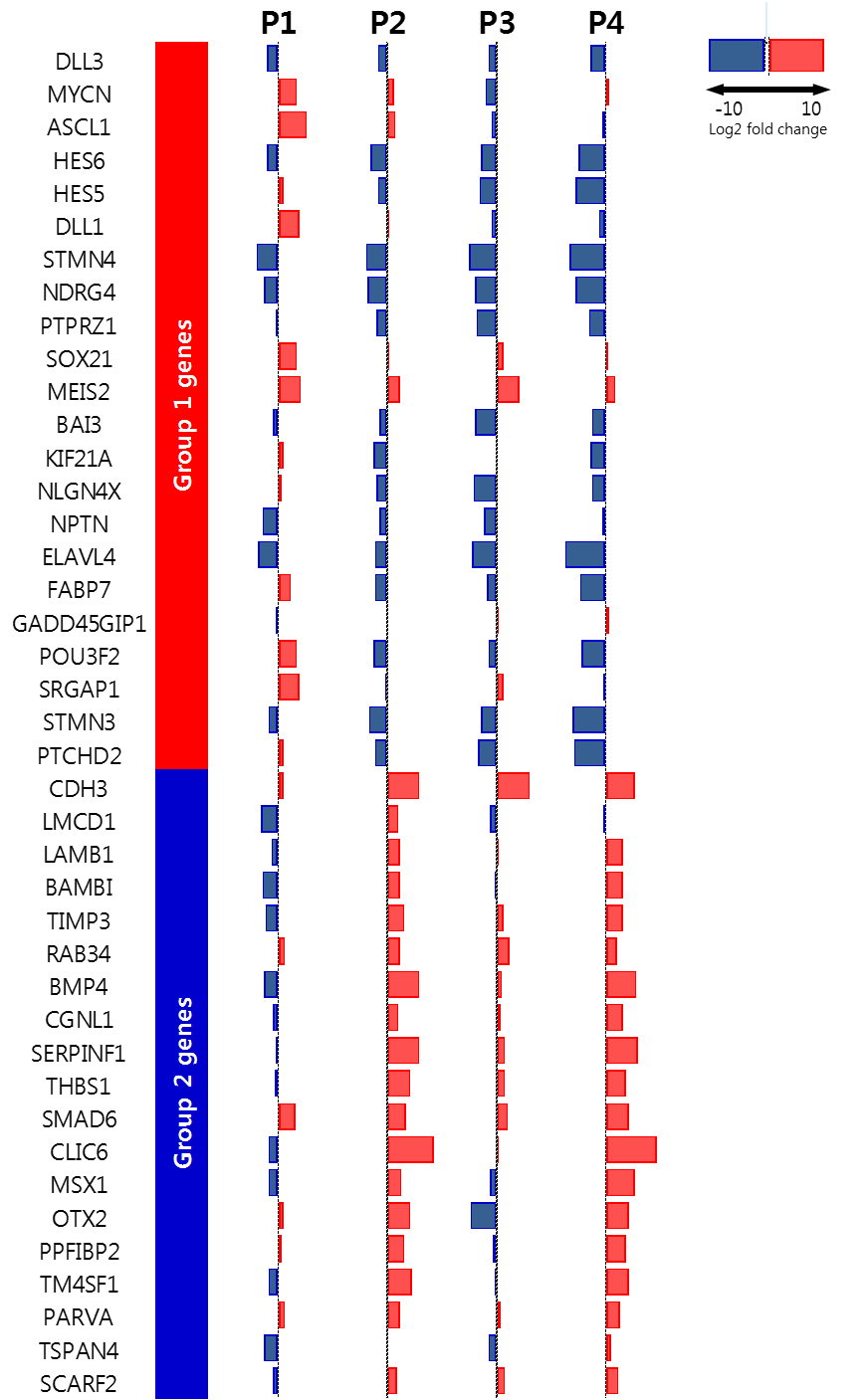


**Figure S7.** NPM1 alteration frequency in TCGA data.


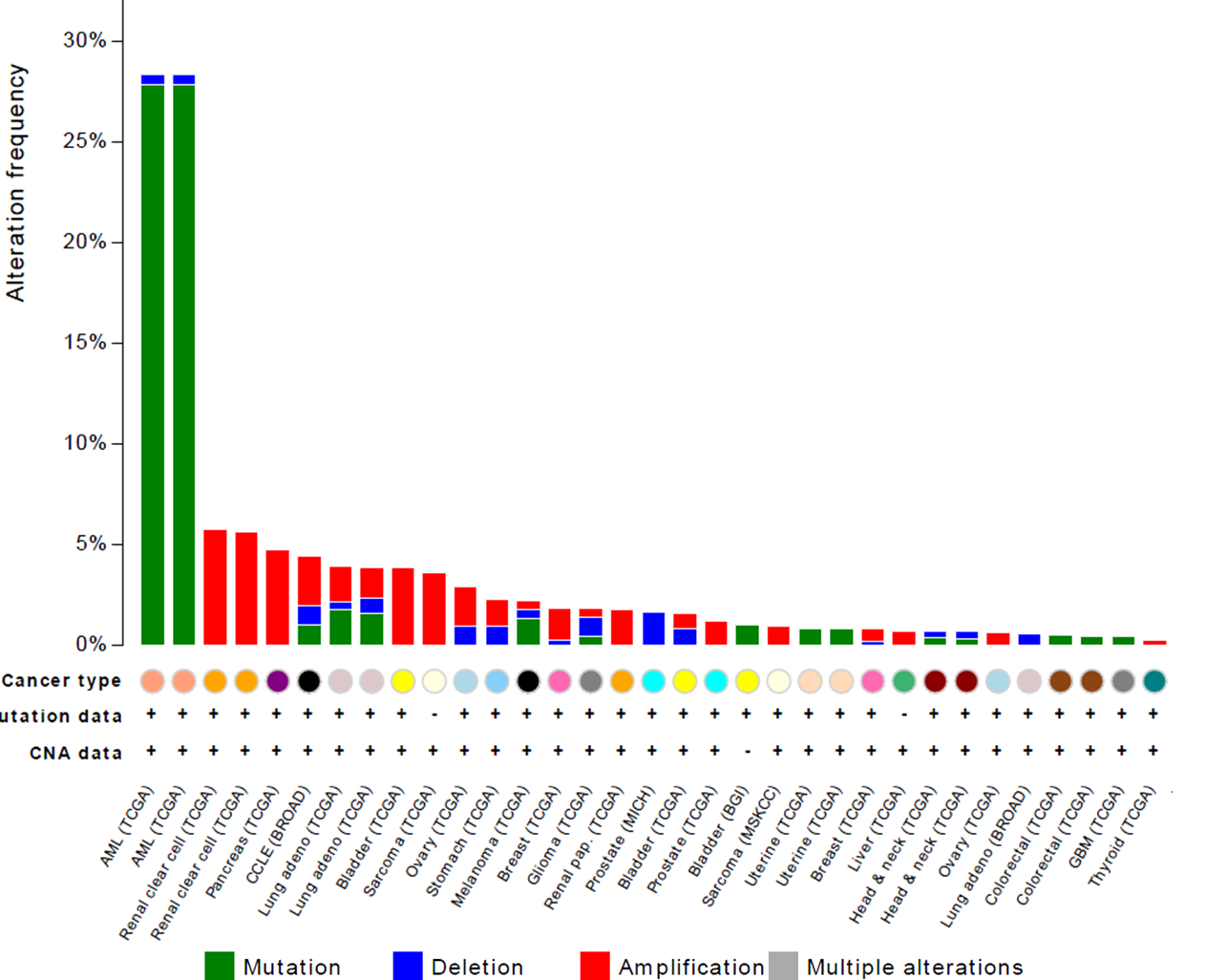


**Figure S8.** Subgrouping of BT-12 and BT-16 cell lines.


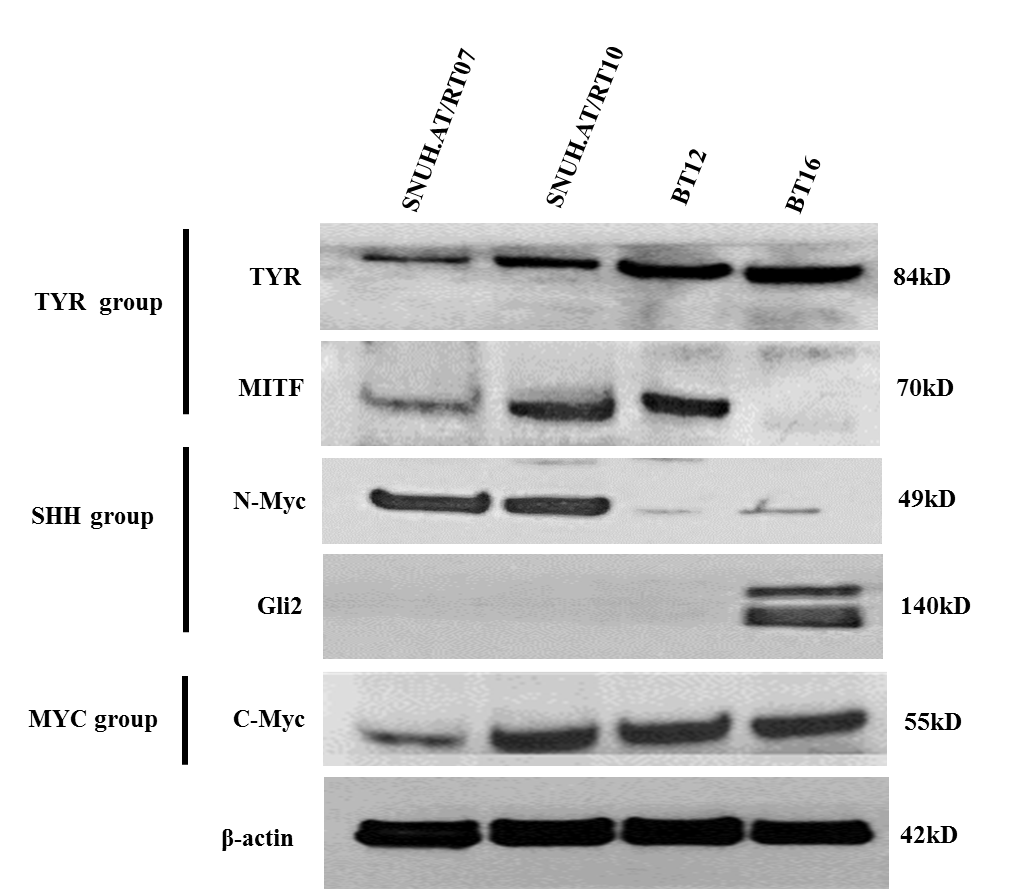


**Reference**

1. Johann PD, Erkek S, Zapatka M, Kerl K, Buchhalter I, et al. (2016) Atypical Teratoid/Rhabdoid Tumors Are Comprised of Three Epigenetic Subgroups with Distinct Enhancer Landscapes. Cancer Cell 29: 379-393.

2. Torchia J, Picard D, Lafay-Cousin L, Hawkins CE, Kim SK, et al. (2015) Molecular subgroups of atypical teratoid rhabdoid tumours in children: an integrated genomic and clinicopathological analysis. Lancet Oncol 16: 569-582.
